# A left ventricular true aneurysm

**DOI:** 10.1002/ccr3.1505

**Published:** 2018-04-06

**Authors:** Yehia Saleh, Abdallah Almaghraby, Ola Abdelkarim, Mahmoud Abdelnaby, Basma Hammad

**Affiliations:** ^1^ Michigan State University East Lansing Michigan; ^2^ Faculty of Medicine Alexandria University Alexandria Egypt; ^3^ Medical Research Institute Alexandria University Egypt

**Keywords:** Acute myocardial infarction, heart failure, left ventricular angiogram, true ventricular aneurysm

## Abstract

True ventricular aneurysm is a scarred wall that most commonly results after an unrevascularized ST elevation myocardial infarction. Patients usually present with heart failure, angina, ventricular arrhythmia, systemic embolization, or ventricular rupture. Diagnosis can be achieved via echocardiography, left ventricle angiogram, cardiac computed tomography, or cardiac magnetic resonance.

A 63‐year‐old gentleman with diabetes presented with dyspnea. Electrocardiography showed Q waves in the inferior leads (Fig. 1). Echocardiography showed a reduced systolic function and a large inferior wall aneurysm (Video S1). Coronary angiography showed three‐vessel disease (Videos [Link], [Link], [Link]). Left ventricular angiography showed a large aneurysm of the inferior wall (Video S5, Fig. 2). As the patient did not have any previous heart disease, we concluded that he developed a silent infarction due to his diabetes and presented later with heart failure. True ventricular aneurysm is a scarred wall that usually results after an unrevascularized ST elevation myocardial infarction; other causes include sarcoidosis, Chagas disease, and hypertrophic cardiomyopathy. Patients usually present with heart failure, angina, ventricular arrhythmia, systemic embolization, or ventricular rupture. Treatment is directed toward heart failure. However, aneurysmal repair is reasonable in patients with intractable ventricular arrhythmias or heart failure unresponsive to medical treatment.

**Figure 1:**
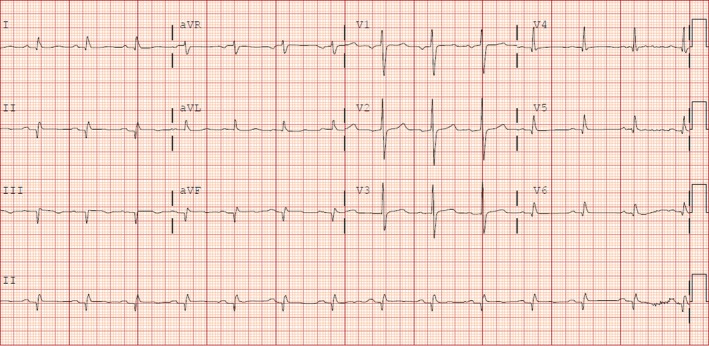
Electrocardiogram showing Q waves in lead II, lead III, and AVF.

**Figure 2:**
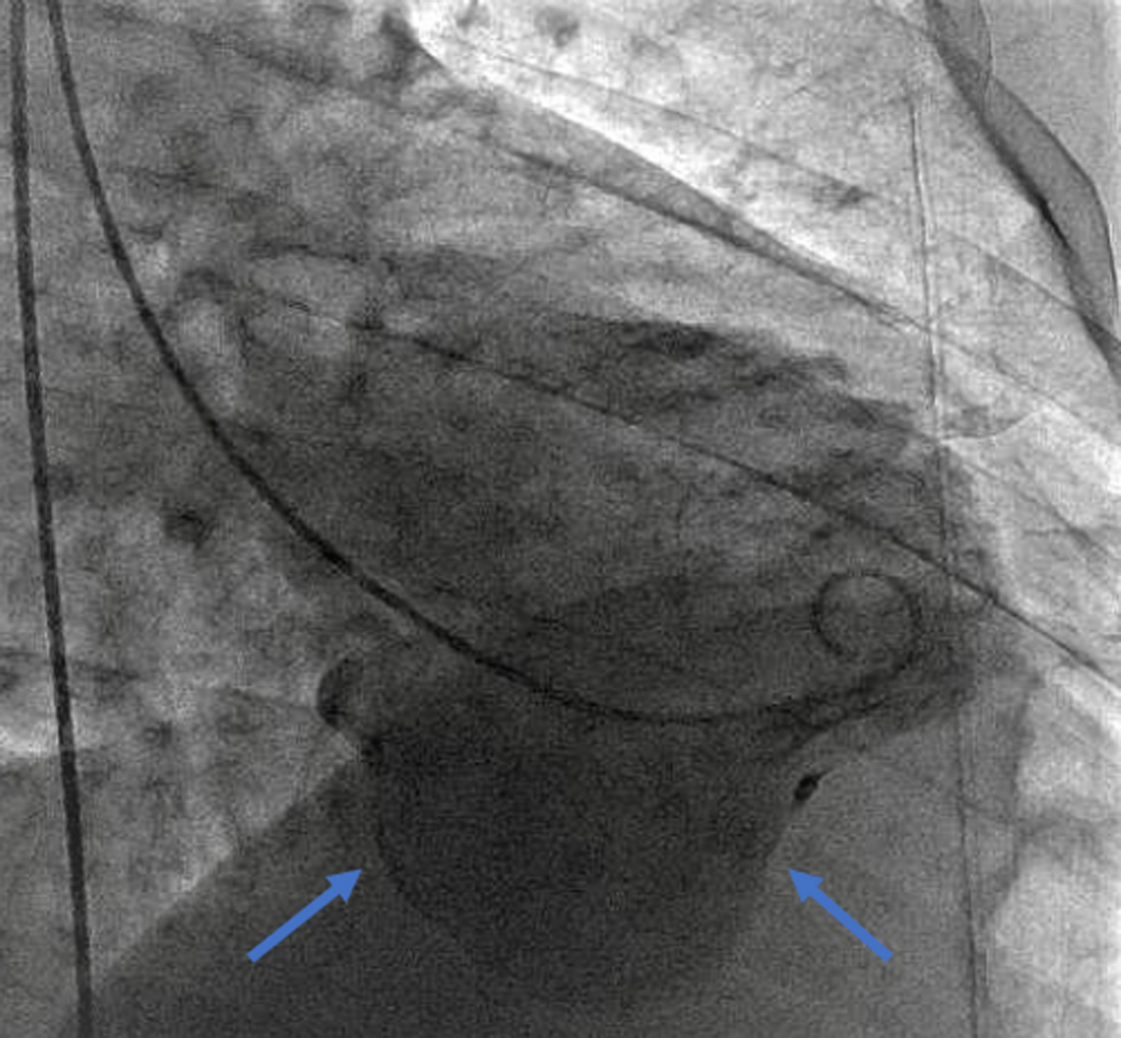
Left ventricular angiogram showing a large inferior wall aneurysm.

## Authorship

YS: involved in management of the patient, wrote the first draft. AA: involved in management of the patient, performed major revision, and approved the final manuscript. OA: involved in management of the patient, approved the final manuscript. MA: involved in management of the patient, approved the final manuscript. BH: involved in management of the patient, approved the final manuscript.

## Conflict of Interest

None declared.

## Supporting information


**Video S1.** Echocardiogram showing reduced systolic function and an inferior wall aneurysm.

**Video S2.** Coronary angiogram showing proximal 90% lesion in the left circumflex artery.

**Video S3.** Coronary angiogram showing 70% lesion in the proximal left anterior descending artery.

**Video S4.** Coronary angiogram showing proximal total occlusion of the right coronary artery.

**Video S5.** Left Ventricular Angiogram showing a large inferior wall aneurysm.

